# When Time Matters: Diagnosis of Percheron Stroke

**DOI:** 10.7759/cureus.78234

**Published:** 2025-01-30

**Authors:** Walid Sadki, Naima Chtaou, Siham Bouchal, Aouatef El Midaoui, Zouhayr Souirti, Mohamed Faouzi Belahsen

**Affiliations:** 1 Department of Neurology, Hassan II University Teaching Hospital, Fez, MAR; 2 Laboratory of Epidemiology and Research in Health Sciences, Sidi Mohamed Ben Abdellah University, Fez, MAR

**Keywords:** artery of percheron, midbrain, paramedian thalamic infarction, thalamus, thrombolysis

## Abstract

The artery of Percheron (AOP) is a rare vascular variant that can cause bilateral paramedian thalamic infarctions when occluded. Due to its atypical clinical presentation and subtle imaging findings, this condition often leads to significant diagnostic challenges. The timely recognition of this condition is critical to implementing appropriate management strategies and improving patient outcomes. We report the case of a 76-year-old man with no prior medical history who presented to the emergency department with a sudden onset of impaired consciousness and vertical gaze palsy. On arrival, the patient was in a state of sleep-like coma. This clinical presentation, combined with the sudden onset of symptoms, was highly suggestive of an AOP infarction. The initial CT findings were unremarkable. Intravenous thrombolysis was administered due to clinical suspicion. The patient demonstrated rapid clinical improvement and regained consciousness in several hours. A follow-up CT scan 24 hours later confirmed bilateral paramedian thalamic infarction, establishing the diagnosis. Although the patient experienced moderate residual cognitive impairment and mild vertical gaze paresis, he regained functional independence within six months. AOP infarction is a rare but serious condition that can present with various symptoms. Its diagnosis can be challenging due to the nonspecific clinical presentation and limitations of conventional imaging techniques in the acute phase, especially in our local context. Early diagnosis and timely intervention, such as intravenous thrombolysis, are crucial to improving functional outcomes.

## Introduction

The thalamus is supplied by perforating branches of the posterior cerebral artery (PCA) and the posterior communicating artery (PcomA) [1]. In 1973, Gerard Percheron was the first person to describe the artery of Percheron (AOP), which is a rare anatomical variant of thalamoperforating arteries that usually arise from a single P1 segment of the posterior cerebral artery [2]. This artery supplies the paramedian thalami and rostral midbrain bilaterally, and its occlusion can cause a bilateral paramedian thalamic infarction with or without midbrain involvement [1].

The prevalence of AOP infarction in the general population is unclear, with estimates ranging from 11.7% in cadaveric studies [3] to as high as 33% in other reports [4]. AOP infarctions are rare, accounting for 0.1-0.3% of all ischemic strokes in one study [1] and 0.1-2% in another [5]. These infarctions also represent 4-18% of thalamic infarctions [1] and can carry a mortality rate of approximately 12% [6].

AOP infarctions are particularly difficult to diagnose due to their diverse and atypical clinical presentation, which can lead to delays or missed diagnoses. Conventional imaging modalities such as CT often fail to reveal ischemic signs in the acute phase. Although brain magnetic resonance imaging (MRI) with diffusion-weighted imaging (DWI) can reveal bilateral thalamic hyperintensities during this phase, it is not always readily accessible in emergency settings. Timely diagnosis and intervention, such as intravenous thrombolysis (IVT), can significantly affect patient outcomes, particularly when clinical signs such as hypersomnia, vertical gaze palsy, and sudden onset of symptoms are present [1]. Given the critical need for early recognition, this case highlights the importance of considering AOP infarction in patients with unexplained impaired consciousness, particularly when CT scans appear normal.

## Case presentation

A 76-year-old man was admitted to the emergency department (ED) with a sudden onset of impaired consciousness. The patient had no significant medical history and was fully ambulant and independent. According to his family, he was sitting in a café when he suddenly collapsed, lost consciousness, and ceased to respond to external stimuli while keeping their eyes closed. On arrival in the ED, his vital signs were as follows: body temperature 37.1°C, capillary blood glucose 1.3 g/l, blood pressure 120/70 mmHg, heart rate 70 beats/minute, and respiratory rate 10-12 breaths/minute with normal respiration. The auscultation of the heart and lungs was unremarkable.

Neurological examination revealed a sleep-like coma, with only a partial response to painful stimuli, after which the patient quickly returned to an unresponsive state, with a Glasgow Coma Scale (GCS) of 7 (E2, V1, M5). No other abnormalities were noted on the initial physical examination. Laboratory workup at admission was normal.

**Figure 1:**
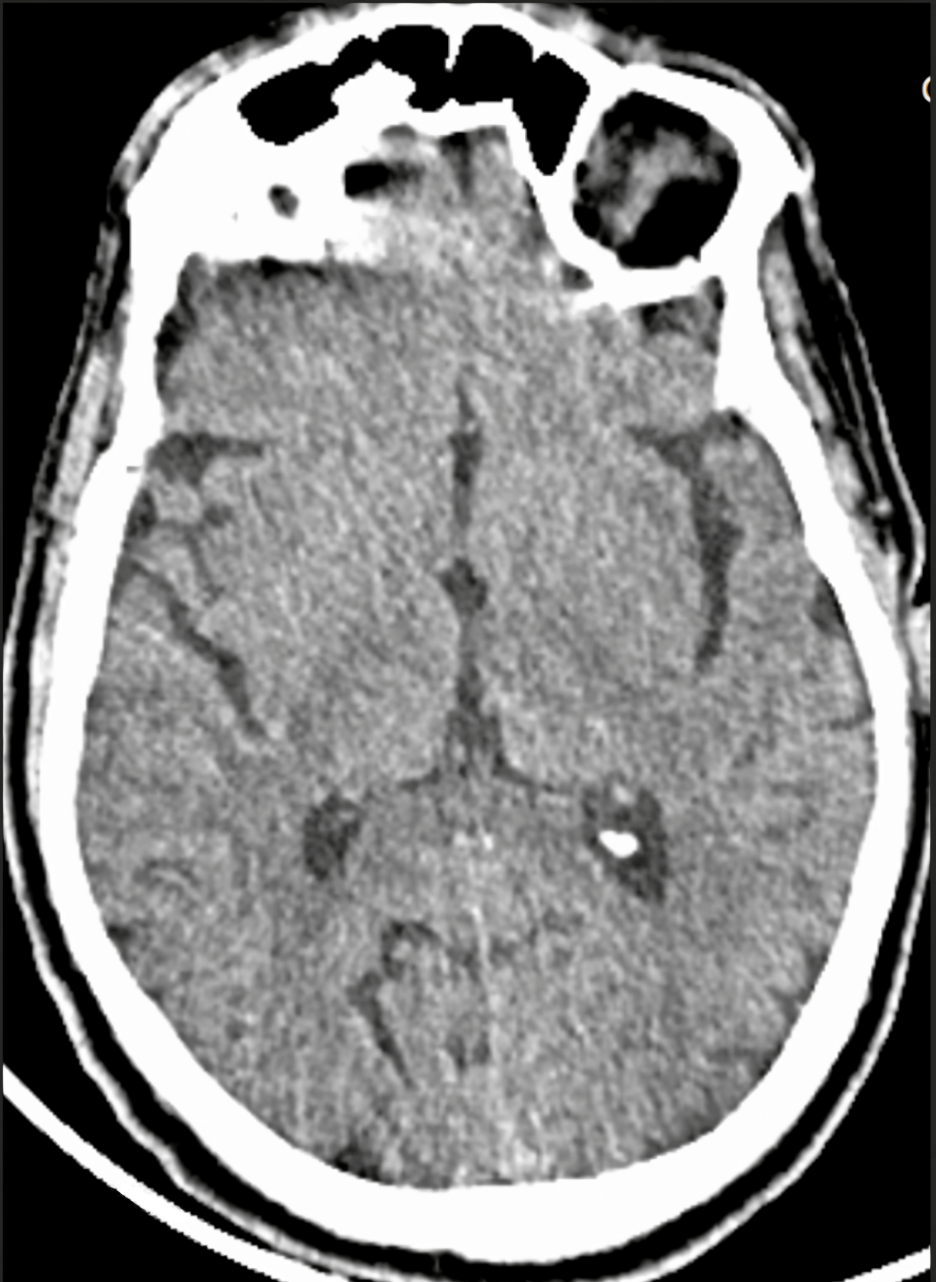
Normal baseline non-enhanced CT scan, without signs of hemorrhage or acute infarction.

**Figure 2:**
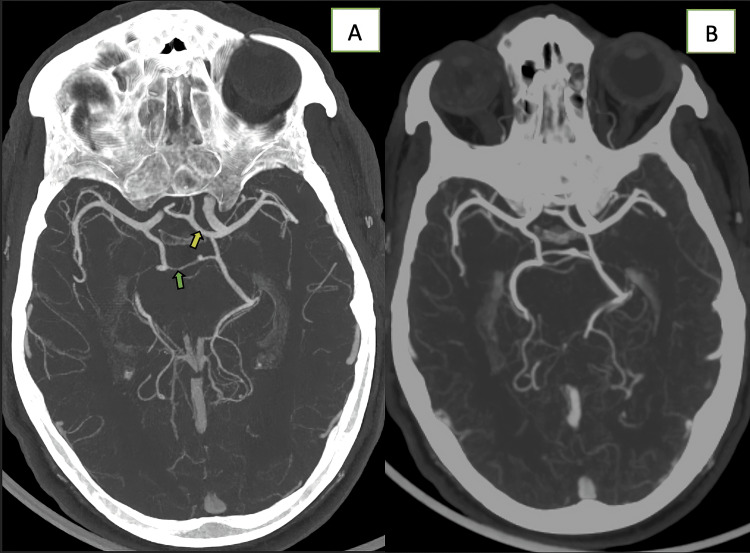
CT angiography of the head and neck does not show any hemodynamically significant stenosis or thrombosis but reveals a fetal-type right PCA (green arrow) and agenesis of the right A1 segment, with both anterior cerebral arteries arising from a single trunk (yellow arrow) (A). The rest of the circle of Willis is normal (B). PCA: posterior cerebral artery

An emergency CT scan performed four hours after the onset was interpreted as normal, without signs of hemorrhage or acute infarction (Figure 1). CT angiography (CTA) of the head and neck did not show hemodynamically significant stenosis or thrombosis, but revealed a fetal-type right posterior cerebral artery and agenesis of the right A1 segment, with the remaining Circle of Willis appearing normal (Figure 2). The electrocardiogram (ECG) showed a normal sinus rhythm.

**Figure 3:**
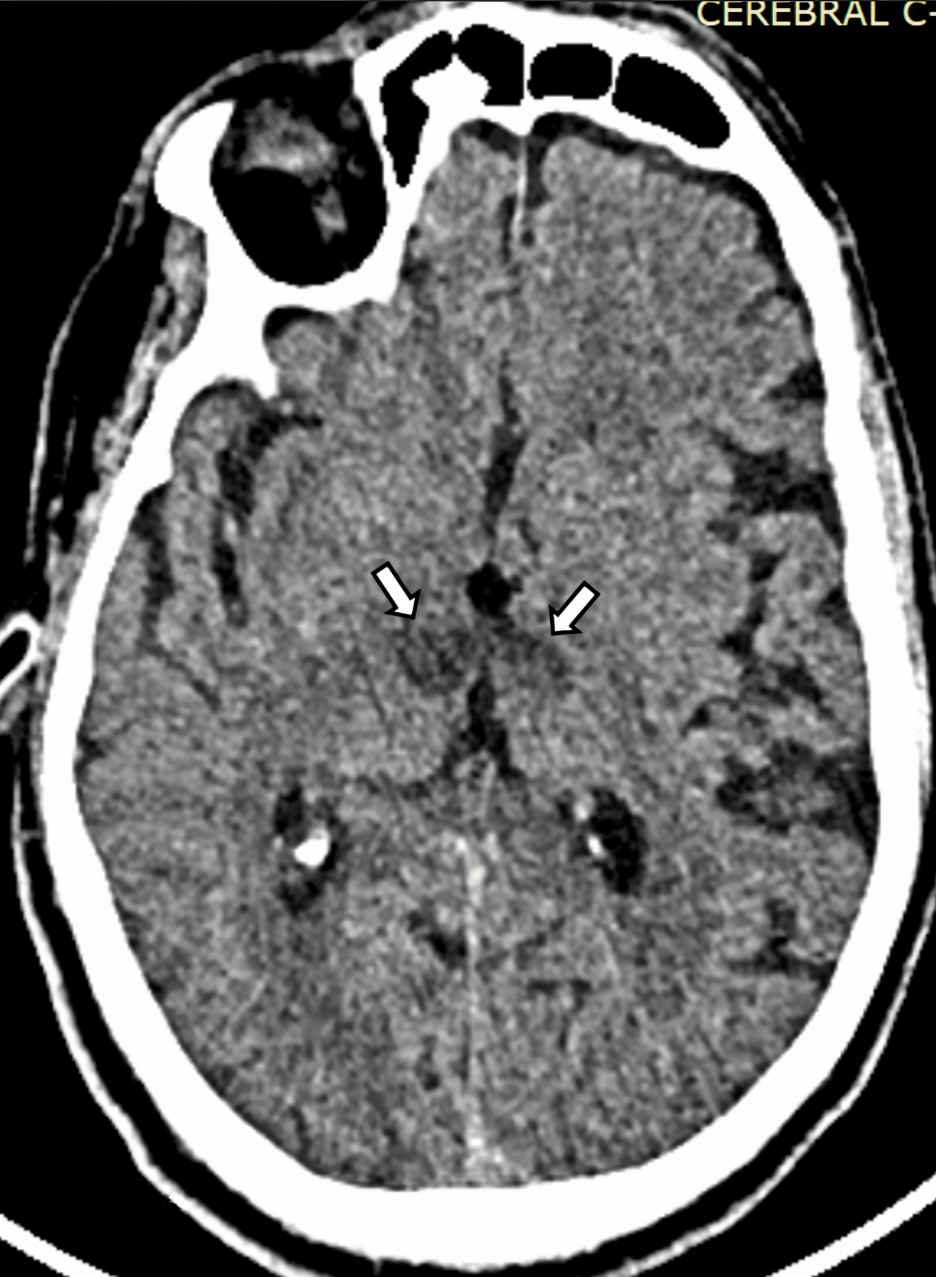
Non-enhanced CT performed 24 hours after IVT revealed bilateral hypodensities in the paramedian thalamic regions (white arrows) without hemorrhagic transformation, confirming the diagnosis of an AOP infarction. AOP: artery of Percheron; IVT: intravenous thrombolysis

Given the highly suggestive clinical context of AOP infarction, IVT with tenecteplase was started four hours and 20 minutes after the onset of the symptom. At 30 minutes post thrombolysis, the patient’s GCS improved to 12 (E4, V3, M5). One hour later, the GCS further increased to 14 (E4, V4, M6), and the patient began following commands. On examination, severely limited upward and downward vertical gaze was observed in both eyes, which was overcome with the doll’s head maneuver. Horizontal gaze remained intact, and the pupils were equal and reactive. On the first day post admission, the patient presented with hypersomnia, disorientation, and moderate memory impairment. A follow-up unenhanced CT scan performed 24 hours after IVT revealed bilateral hypodensities in the paramedian thalamic regions without hemorrhagic transformation, confirming the diagnosis of AOP infarction (Figure 3).

**Figure 4:**
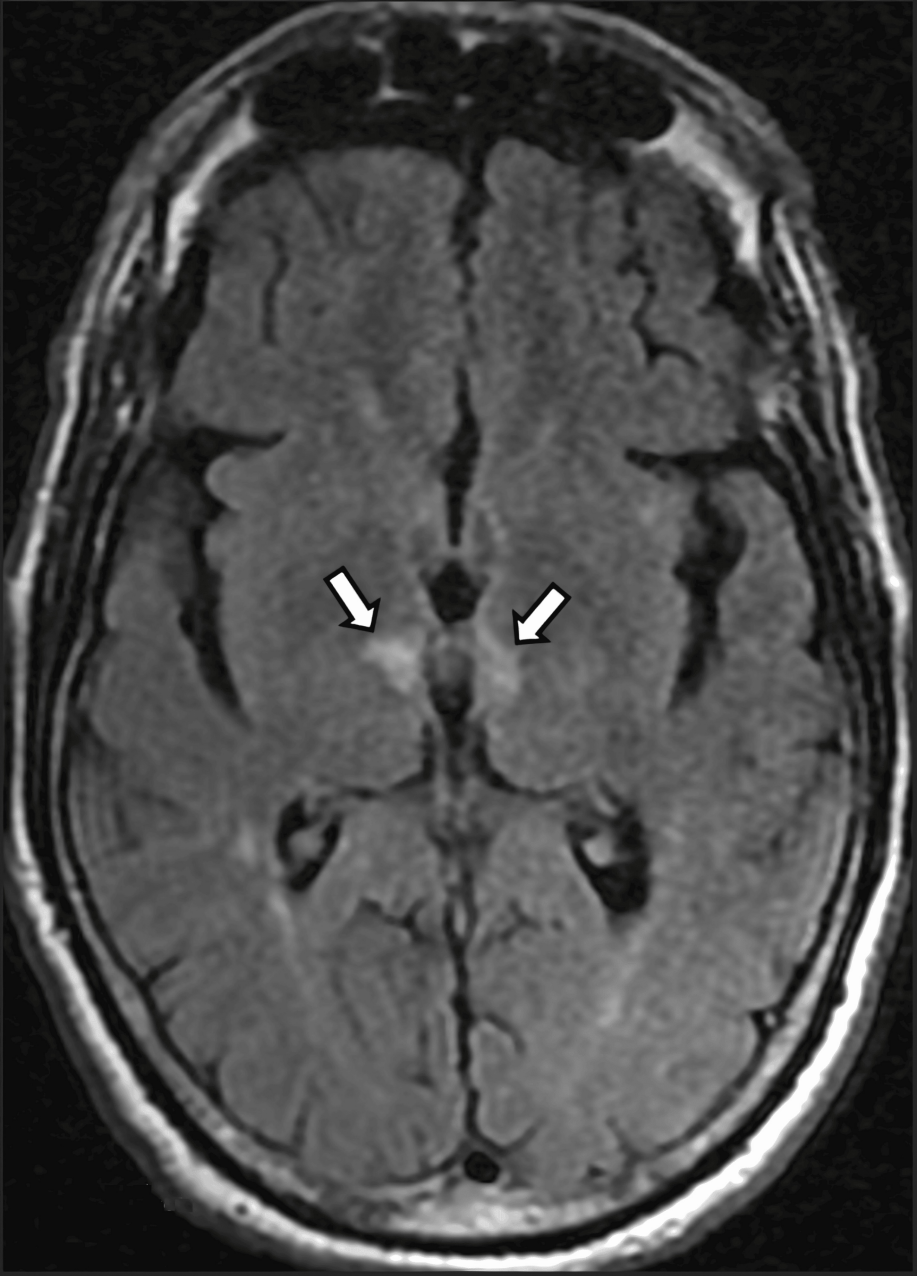
Bilateral paramedian thalamic hyperintensities in FLAIR sequences (white arrows) were consistent with the diagnosis of AOP infarction. AOP: artery of Percheron; FLAIR: fluid-attenuated inversion recovery

Over the next seven days, the patient gradually improved, with hypersomnolence and disorientation resolving by day 7. His clinical condition further improved, and at the six-month follow-up, he achieved functional independence with a modified Rankin Scale score of 2. Three months after symptom onset, downward gaze was restored, although mild limitation of upward gaze remained. Concurrently, Mini-Mental State Examination (MMSE) scores were 22/30 at one month, 25/30 at three months, and 26/30 at six months. Despite these cognitive improvements, the patient continued to exhibit moderate impairments in attention and calculation, alongside mild deficits in working memory and mental planning. Furthermore, the patient underwent a follow-up MRI 40 days after symptom onset, which revealed bilateral paramedian thalamic hyperintensities in fluid-attenuated inversion recovery (FLAIR) sequences, consistent with the diagnosis of AOP infarction, without additional ischemic lesions (Figure 4).

The only notable finding in the stroke etiology examination was elevated low-density lipoprotein (LDL) cholesterol (0.9 g/L) and a stable, non-stenotic atherosclerotic plaque in the left primary carotid artery. All other investigations, including transthoracic echocardiography (TTE), Holter ECG, transcranial Doppler for right-to-left shunt, and blood tests were unremarkable. Cerebral MRI did not show any evidence of microangiopathy. During hospitalization, the patient's blood pressure remained stable and the ECG monitoring was uneventful. The patient was treated with a dual antiplatelet regimen for 21 days, statins, and fluoxetine followed by a single antiplatelet regimen (aspirin). We concluded with a cryptogenic ischemic infarction of AOP.

## Discussion

Challenging clinical presentation

The clinical manifestations of AOP infarction are diverse and can be nonspecific at onset, with common presentations including altered consciousness and vertical gaze palsy [5,7]. Our patient exhibited these symptoms, which raised the suspicion of AOP infarction. Altered mental status can occur anywhere in the spectrum, from drowsiness or confusion to hypersomnolence or coma [5].

Particular emphasis was placed on a clinical presentation, as observed in our case, characterized by a sleep-like coma with minimal or absent neurological signs, which may be misleading and potentially direct clinicians to alternative diagnoses such as metabolic or toxic causes [8]. Sleep-like coma can manifest in two forms: a mild, brief, and easily arousable state or a deeper, prolonged state that requires painful stimuli, as seen in our case [8]. Vertical gaze palsies typically indicate midbrain involvement, but they have also been observed in patients without midbrain lesions, as observed in our patient. This phenomenon may be explained by the disruption of cortical inputs that pass through the thalamus on their way to the rostral interstitial medial longitudinal fasciculus [9].

In addition to the classical triad of altered mental status, vertical gaze palsy, and amnesia, its clinical presentation can lead to other oculomotor disorders, hemiplegia, cerebellar ataxia, and movement disorders.

Challenges in diagnostic imaging

The diagnosis of AOP infarction remains a considerable challenge owing to its rarity and the subtlety of the imaging findings, particularly in the acute phase. Similar to our case, CT imaging often appears normal initially, and typical bi-thalamic hypodensities may only become evident several hours after the onset of the symptom, resulting in a delayed diagnosis [5,10]. MRI significantly improves detection through characteristic bilateral paramedian thalamic hyperintensities in T2-weighted, FLAIR, and DWI, accompanied by a reduction in the apparent diffusion coefficient (ADC) [5]. However, the variable involvement of associated structures, such as the upper midbrain and anterior thalamic nuclei, and the limited availability of MRI in emergency settings can further hinder timely identification [11]. The latency between the clinical onset of AOP infarction and its full manifestation on DWI sequences is not well-established [10]. In addition, early findings of AOP infarction may be absent on initial MRI, even in DWI sequences [12,13]. The AOP is infrequently visualized using conventional angiography and is below the resolution limits of both CTA and magnetic resonance angiography (MRA) [14].

Challenging time window to treatment

Prompt diagnosis and intervention are critical for optimizing outcomes in patients with AOP infarction. In this particular case, timely administration of IVT resulted in marked clinical improvement. Despite notable recovery, the patient continued to demonstrate mild vertical gaze paresis and persistent cognitive deficits.

Thrombolytic therapy, when administered within 4.5-6 hours of symptom onset, aims to achieve recanalization [15]. Thrombolysis remains a primary treatment strategy for acute AOP infarction, with the potential for complete recovery or mild residual deficits at follow-up [11,16]. Conversely, conservative management has also demonstrated favorable outcomes in a subset of patients [16].

The selection of therapeutic strategy and subsequent management for AOP infarction is guided by the underlying etiology, most frequently cardioembolism or small-vessel disease [17,18], underscoring the importance of tailoring treatment decisions to each patient’s unique clinical context.

## Conclusions

The AOP infarction poses a significant diagnostic challenge, often missing on initial imaging and being confused with other causes of altered consciousness. Early recognition and timely intervention, such as IVT, may be essential to improve clinical outcomes and prevent unnecessary procedures, although some cognitive deficits may persist. This case emphasizes the importance of considering AOP infarction in patients with a rapid onset of altered consciousness, particularly when typical findings such as bilateral thalamic hyperintensity on DWI are not immediately available in emergency settings and conventional CT scans appear to be normal.
